# Therapeutic interventions for heart failure in Colombia: result of a Delphi panel

**DOI:** 10.1371/journal.pone.0304124

**Published:** 2024-09-03

**Authors:** Clímaco de Jesús Pérez Molina, Carlos Castañeda Orjuela, Pedro Valbuena Hernandez, Rafael Ignacio Pérez Arias, María Alejandra Pérez Arias, Ana María Arias Copete

**Affiliations:** 1 Department of Cardiology, Clinic of The Universidad de la Sabana, Bogotá, Colombia; 2 Electrophysiology Service, Sanitas Colombia Clinic, Bogotá, Colombia; 3 AVIDANTI Clinic, Ibagué, Colombia; 4 National Health Observatory, Bogotá, Colombia; 5 Center for Economy Research Universidad del Bosque, Bogotá, Colombia; 6 Medical Student at Rosario University, Bogotá, Colombia; 7 Medical Student of Sabana University, Chía, Colombia; 8 Breast Cancer Surgery Coordinator, Nogales Clinic, and Colsanitas, Bogota, Colombia; University of Sharjah, UNITED ARAB EMIRATES

## Abstract

**Objective:**

The objective of this study was to validate the main therapies used in the treatment of heart failure through a clinical consensus conducted by cardiology experts in Colombia.

**Methods:**

The Delphi technique was employed, which involves a series of consultation rounds with experts to reach a consensus. Cardiologists with experience in HF treatment were selected, and they were sent electronic questionnaires to assess the relevance of various therapeutic interventions. Consensus was defined when at least 70% of the experts agreed on the relevance of an intervention.

**Results:**

Fourteen cardiology experts participated in the study. In the first round, nine therapeutic interventions were evaluated, but insufficient agreement was reached to form a consensus. A second round was conducted, where feedback was provided to the experts, and they were asked to rate the relevance of the interventions using a Likert scale. Consensus was achieved for eight of the evaluated therapeutic interventions. The focus of the third round was on the interventions that had not reached consensus in the previous rounds.

**Conclusions:**

This study provides clinical consensus on therapeutic interventions for HF in Colombia. Nine therapeutic interventions were identified as relevant by the experts. These findings can help improve HF treatment and optimize clinical outcomes in Colombia. It is important to note that this study was conducted with local experts, and the results may not be generalizable to other populations.

## Introduction

Heart failure (HF) is a heterogeneous and complex syndrome characterized by clinical manifestations such as dyspnea, ankle swelling, and fatigue, accompanied by signs such as elevated jugular venous pressure, pulmonary crackles, and peripheral edema [1]. These symptoms and signs are caused by functional deficiencies and self-regulatory mechanisms of cardiovascular physiological mechanics, resulting in tissue hypoperfusion, congestion, and venous overflow at the systemic level. At the cardiac level, it leads to alterations in the five basic functions of the heart: chronotropy, inotropy, bathmotropism, lucitropism, and dromotropism. It leads to increased ventricular filling pressure both at rest and during stress [1]. Given these characteristics, heart failure (HF) requires for its treatment: Non-Pharmacological Treatment (NPT), Optimal Pharmacological Treatment (OPT), Cardiac Resynchronization Therapy, Cardiac Resynchronization, Implantable Cardioverter Defibrillator (ICD), Cardiac Surgery in HF (Surgery), Intravenous Treatment for Advanced HF (IV Treatment), Mechanical Circulatory Support Device (MCSD), and Heart Transplantation (HT).

Each year, approximately 20% of all HF patients are hospitalized, making it one of the main causes of hospitalization and imposing a significant economic burden on healthcare systems. The healthcare cost associated with HF patients is estimated to represent between 1% and 3% of total healthcare expenditure in North America, Latin America, and Europe [2, 3]. Additionally, it is estimated that there are currently around 26 million adults living with HF worldwide, and this number is expected to increase by the year 2030 [2]. National epidemiological records show a current prevalence of HF ranging from 1% to 2%, which increases to over 10% in individuals aged 70 or older. Furthermore, the prevalence of HF is expected to continue rising due to population aging and the increase in comorbidities [2, 3]. Therefore, HF will increasingly become one of the most common diseases in the elderly population.

On the other hand, HF is the end result of many cardiac disorders, and its incidence is expected to exponentially increase in the future [1]. Although there are available therapies that have shown to improve mortality and morbidity associated with HF [2–5], these therapies are often underutilized or not prescribed correctly [6, 7]. Treatment optimization has been shown to reduce hospitalizations and treatment costs [8–10].

Nevertheless, cardiologists are responsible for accurate diagnosis and follow-up of patients with chronic HF [1]. Despite the existence of updated management guidelines for HF, symptoms that could suggest HF are often unrecognized or confused with other diagnoses [9]. This has led to underdiagnosis of HF, as evidenced by high prevalence rates (representing up to 80% of all HF cases) in high-risk community populations. Furthermore, the incorporation of scientific knowledge included in these guidelines into clinical practice may be slow or lacking sufficient validation by experts. Smeet et al. (2019) concluded that a shift towards a more comprehensive risk assessment is needed, including access to tests that allow for a validated diagnosis of HF [8, 10]. Based on these considerations, this analysis has been designed with the aim of validating, through a clinical consensus based on a Delphi panel, the relevance of the main therapies in the treatment of HF among expert cardiologists.

## Methods

The Delphi panel is a structured interactive technique used to develop consensus or near-consensus among experts on the elements that should be included in a study or tool [11, 12]. In this case, the purpose of the panel is to evaluate therapeutic interventions for HF based on the level of agreement or consensus. To be designated as an expert in this study, participants had to meet specific criteria: 1) certification as cardiologists with training in HF, covering cases both with and without the risk of sudden cardiac death (SCD), as well as practical experience in treating HF patients; and 2) certification as interventional cardiologists with training in HF, including cases with and without the risk of SCD, and experience in managing such patients. Furthermore, these experts had to practice in selected clinics and hospitals: Clínica Colombia de Sanitas, Hospital del Tunal, Clínica Medical Proinfo in Bogotá DC, Clínica de la Universidad de la Sabana in Chía, near Bogotá, Clínica Meta in Villavicencio, and Clínica Avidanti in Ibagué. All these institutions are part of the hospital network in the central Andes region of Colombia. Consensus was established when at least 70% of the expert’s reached agreement on an intervention [13]. Additionally, starting from the second round of the panel, the author could expand responses, clarify doubts to reach agreement, or request other treatments that were not initially considered.

### Selection of therapeutic interventions and definition of consensus

For this study, 9 therapeutic interventions with the strongest evidence were initially identified through a literature review conducted previously. This initial list of interventions was formulated based on American and European guidelines for the management and treatment of HF [14, 15]. They are described below:

1. Optimal Pharmacological Treatment (OPT): This refers to the administration of medications following current clinical guidelines for HF management. It includes the use of medications such as angiotensin-converting enzyme inhibitors (ACEIs), angiotensin II receptor blockers (ARBs), beta-blockers, diuretics, and other agents aimed at alleviating symptoms and improving cardiac function in HF patients.
2. Non-Pharmacological Treatment (NPT): This term denotes therapeutic strategies and approaches that do not involve medications. It includes lifestyle adjustments such as low-sodium diets, supervised physical activity, stress management, moderation in alcohol consumption, and smoking cessation. Furthermore, non-pharmacological treatment includes interventions like patient education, comorbidity management, and cardiac rehabilitation therapies.
3. Cardiac Resynchronization Therapy with Defibrillator (CRT-D): This therapy involves the implantation of a medical device that uses electrical stimulation to improve the synchronization of cardiac contractions. When necessary, it provides defibrillation to address potentially life-threatening ventricular arrhythmias.
4. Cardiac Resynchronization Therapy without Defibrillator (CRT-P): Similar to CRT-D, this therapy aims to synchronize cardiac contractions through electrical stimulation but does not include defibrillation. It is used in HF patients who do not face a substantial risk of potentially life-threatening arrhythmias.
5. Implantable Cardioverter Defibrillator (ICD): This is a medical device implanted in the body to continuously monitor heart rhythm and provide rapid and effective defibrillation therapy in response to potentially life-threatening ventricular arrhythmias such as ventricular fibrillation.
6. Cardiac Surgery in HF (Surgery): This category encompasses various surgical procedures designed to treat or improve conditions associated with HF. It includes coronary artery bypass surgery, valve replacement, repair of congenital heart defects, and, in severe cases, heart transplantation.
7. Intravenous Treatment for Advanced HF (IV Treatment): This involves the administration of medications directly into the bloodstream through intravenous access to achieve faster and more effective management of acute or severe HF. Intravenous medications may include diuretics, positive inotropic agents, or vasodilators.
8. Mechanical Circulatory Support Device (MCSD): These devices serve as a bridge to heart transplantation or provide long-term support for patients with advanced HF. They can be left ventricular, right ventricular, or biventricular and are designed to improve blood circulation in the heart.
9. Heart Transplantation (HT): This procedure involves replacing the dysfunctional heart of a patient with a healthy one from a compatible donor. It is reserved for cases of advanced HF when alternative treatments have not been effective or have been exhausted.

### First round of the Delphi panel

In the first round, the human resources department of each institution sent a written request to the head of the cardiology department, who then provided feedback to their team. This facilitated the identification of cardiologists in each institution who met the selection criteria. Cardiologists who met these criteria were contacted via the email addresses provided by their respective institutions. After receiving a detailed explanation of the data collection process, those who agreed to participate in the study and signed informed consent were asked to provide their phone numbers and WhatsApp contacts to facilitate communication. Subsequently, a text message containing a link to an electronic questionnaire, created using Google Forms, was sent to the participants. This first round lasted one week, with reminders sent to non-responders after one or two days. Additionally, the first phone call was made between the third and fifth day, followed by a second call after the sixth day. The results obtained in this round were shared with all participants.

### Second round of the Delphi panel

Participants were provided with a link to a second electronic questionnaire, also generated using Google Forms, and distributed via WhatsApp. In this questionnaire, respondents were asked to rate their agreement with the questioned therapeutic intervention for the treatment of HF using a Likert scale from 1 to 10. A score of 1 indicated "totally disagree," 2 represented "disagree," 3 indicated "somewhat disagree," 4 denoted "slightly disagree," 5 meant "neutral," 6 represented "somewhat agree," 7 indicated "almost agree," 8 denoted "agree," 9 indicated "strongly agree," and 10 meant "totally agree." Furthermore, the second round introduced an option for other therapies through four new questions. Two of these questions asked for therapeutic interventions that panelists considered necessary but were not initially considered by the author, both for HF with and without the risk of SCD. There was no limit to the number of elements they could propose, and they were also encouraged to provide comments. The other two questions focused on breaking down pharmacological treatments for HF with and without the risk of SCD, as described in the literature. As a result of these questions, two new interventions were introduced: "other interventions" and pharmacological treatment for SCD, increasing the initial number of interventions from 9 to 11. This round was given more time, one week, with reminders sent similarly to the first round. Feedback on the results was provided to the panelists at the end of this round.

### Third round of the Delphi panel

To avoid participant fatigue, a third round was conducted, focusing solely on interventions that had not reached consensus in previous rounds. These interventions were divided into two parts, A and B, related to the indication for CRT-P implantation in individuals over 80 years old and in those with atrioventricular block and a certain degree of HF, respectively. Each expert was asked to reevaluate their ratings considering the opinions of others, allowing them to maintain or modify their responses as they saw fit. Additionally, all participants were asked to provide reasons for any changes in their opinions. The primary goal of this third round was to improve the convergence of opinions and establish consensus. Like the previous rounds, this third round lasted one week, and feedback based on its results was provided.

### Statistical analysis

Responses from participants were collected in Microsoft Excel and analyzed using STATA 15.0 and SPSS. To evaluate the most significant variables within the model and validate the technique’s instrumentation, the standard error was used. The degree of agreement between responses was assessed using the Kendall tau coefficient, with a threshold value of 0.9 or higher [16–18]. To examine differences in experts’ opinions on specific questions, the McNemar Bowker test was employed. As with the other rounds, a summary of the results was shared with each expert at the end of this round. Supporting information is available in the appendix at the end of the manuscript. This study received approval from the Research and Ethics Committee of the University of El Bosque.

## Results

Fourteen panelists, all affiliated with selected institutions, participated in this study. Among them, some specialized in performing surgical, percutaneous, or non-invasive interventions (such as echocardiographers, hemodynamic specialists, or electrophysiologists) for patients with HF with or without the risk of SCD, while others were clinicians (cardiologists or specialists) responsible for the ongoing care of these patients. Table 1 presents the main characteristics of the experts who participated in the three rounds of the Delphi process.

**Table 1:** Attributes of the 14 panelists.

Attribute	No. (%) of panelists
Gender	
Menu	12 (85.7%)
Women	2 (14.3%)
Training	
Cardiology	6 (42.8%)
Electrophysiology	3 (21.4%)
Hemodynamics	2 (14.3%)
Echocardiographer	2 (14.3%)
Heart failure	1 (7.2%)
Practice Place	
Private Hospital / Clinic	11 (78.6%)
Teaching hospital	3 (21.4%)
Practice city	
Bogota D.C.	9 (62.9%)
Outskirts Bogotá	3 (21.4%)
Villavicencio	1 (7.2%)
Ibagué	1 (7.2%)
Years of experience caring for patients with Heart Failure	
<1	0 (0)
1–4 years	2 (14.3%)
5–9 years	3 (21.4%)
10–15 years	5 (35.6%)
>15	4 (28.7%)
No. of HF patients seen per month	
<10	0 (0)
10–19 months	0 (0)
20–29 months	2 (14.3%)
30–39 months	6 (42.8%)
>40	6 (42.8%)

N = 14. Created by the author.

After the first round, the responses were subjected to statistical analysis using Kendall’s concordance coefficient (W) to assess agreement between the nine therapeutic interventions for HF. Table 2 provides a summary of the panelists’ responses to the dichotomous questions in the first questionnaire. Kendall’s coefficient (W) yielded a value of r (14) = 0.071, with a significance level of 0.433, an effect size of 0.27, and a power of 0.61. Despite the fact that the value of W indicated a fair agreement, the achieved concordance falls below the expected range of 0.8–1.0 for the test in this study. These findings allow for partial generalization of the sample data to the population. This indicates the need for a second round.

**Table 2:** Delphi survey results (first round).

therapeutic interventions	Consensus (0–100%)
OPT	100%
NPT	100%
ICD	100%
CRTp	100%
CRTd	92.86%
Surgery	100%
IV Treatment	100%
LVAD	100%
Cardiac transplant	100%

Optimal pharmacological treatment (OPT), Non-pharmacological treatment (NPT), Implantable cardioverter-defibrillator (ICD), Resynchronization therapy (CRTp), Resynchronization therapy with a defibrillator (CRTd), Surgery for HF (Cx), Intravenous therapy for HF (IV treatment), Cardiac mechanical assistance device (LVAD), Cardiac transplant (CT). Created by the author.

In the second round, panelists were asked to rate the degree of consensus for each of the therapeutic interventions using the Likert scale, and the results are detailed in Table 3. Regarding the 4 questions regarding proposing other therapies for HF with and without the risk of SCD, and the breakdown of pharmacological treatments for HF and the risk SCD. Regarding medical treatment for SCD, half of the experts (50%) advocated for the use of beta-blockers, while the other half mentioned both beta-blockers and amiodarone (see Table 4).

**Table 3:** Degree of consensus for each of the therapeutic interventions (second round).

Therapeutic Interventions	Panelists	means of the responses	Confidence interval, 95%
OPT	14	9,57	9.27–9.87
NPT	14	9,29	8.87–9.70
ICD	14	8,36	7.17–9.55
CRTd	14	9,36	8.99–9.72
CRTp	14	6,57	4.55–8.59
Surgery	14	8,07	7.05–9.10
IV treatment	14	8,00	7.15–8.85
LVAD	14	8,50	7.52–9.48
CT	14	9,00	8.55–9.45

Optimal pharmacological treatment (OPT), Non-pharmacological treatment (TNF), Implantable cardioverter-defibrillator (ICD), Resynchronization therapy (CRTp), Resynchronization therapy with a defibrillator (CRTd), Surgery for HF (Cx), Intravenous therapy for HF (IV treatment), Cardiac mechanical assistance device (LVAD), Cardiac transplant (CT). Created by the author.

**Table 4:** Additional therapies suggested by the experts, second-round results.

Heart Failure, No. (%)	Risk of sudden death, No. (%)
Patient education, lifestyle changes, 1 (7.2%)	None, 6 (42.8%)
Diet, 1 (7.2%)	Beta blockers, 1 (7.2%)
Cardiac rehabilitation, 2 (14.3%)	Modulation of arrhythmic substrates and sympathectomy, 3 (21.4%)
Iron IV, #3 (21.4%)	
None, 3 (21.4%)	RVC QX Stitch study, 1 (7.2%)
6-Hydralazine and vericiguat, 2 (14.3%)	Ablation, gene therapy, 1 (7.2%)
modulation of arrhythmic substrates, 1 (7.2%)	Rehabilitation, 1 (7.2%)
SGLT2 inhibitors, 1 (7.2%)	None, 1 (7.2%)
Renal denervation and stem cells, 1 (7.2%)	

Intravenous Iron Therapy (Iron IV), Sodium-Glucose Cotransporter 2 (SGLT2) Inhibitors, Surgical Revascularization (RVC QX), Created by the author.

**Fig 1:**
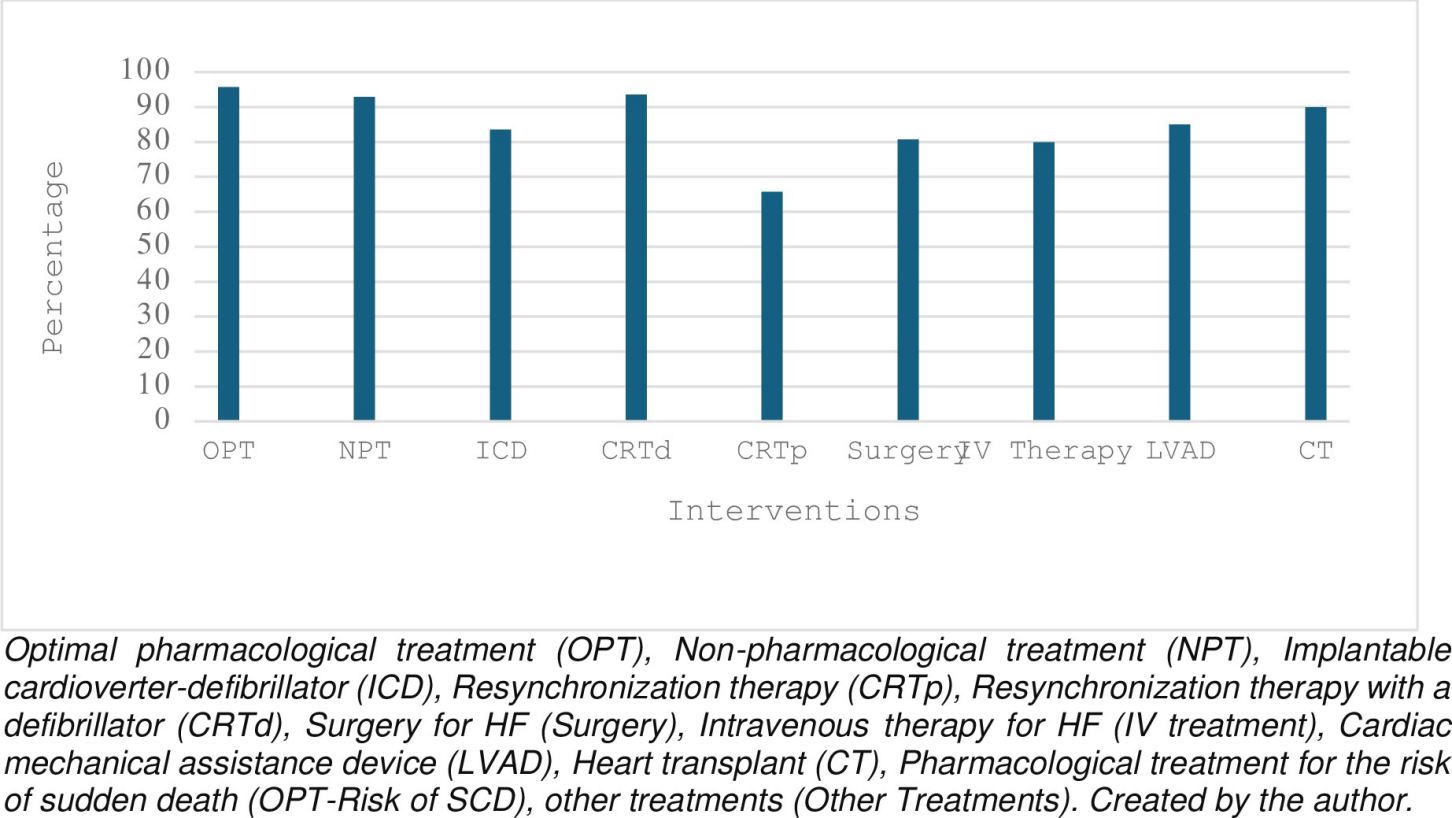
Percentage of consensus on the interventions.

Fig 1 shows the ratings given by experts for the 9 items evaluated in the second round. Consensus was reached for 8 items (90.9%). It is important to note that the most significant variables within the model were OPT, CRTd, NPT, and CT, as a procedure aimed at validating the technique’s instrumentation.

In the third round of the panel, regarding questions A and B about CRTp, five experts changed their initial response from the second round, as shown in Table 5.

**Table 5:** Third round of the CRTp in dissenting experts.

Expert	Initial response	Answer A	Answer B
one	0	6	6
two	1	7	7
three	4	7	7
four	4	7	7
five	3	6	6

*Answer to the first part of the question on CRTp in the third round (Answer*, part A), *Answer to the second part of the question on CRTp in the third round (Answer*, *part B)*. *Created by the author*.

Despite the initial variations, the results indicate that after deliberation and the three rounds of the panel, there was alignment in consensus regarding the CRTp intervention. The average score for CRTp in this round was 8, with a standard deviation of 1.57, reflecting a substantial level of agreement among the experts. The minimum score was 6, and the maximum was 10, highlighting a narrow range of responses, as shown in Table 6. The response rate for this third round was 100%, as all 14 experts completed the questionnaire. This high response rate demonstrates the commitment and participation of the panel of experts in achieving a consensus on therapeutic interventions for HF.

**Table 6:** Statistical measures third round of the CRTp in dissenting experts.

Variable	Obs	Half	%	StDev	Min	Max	Variance	CI, 95%	Err Std
**CRTp**	14	8	80	1.57	6	10	2.46	7,094–8,905	0.419

Observation (Obs) Standard Deviation (StDev), Minimum (Min), Maximum (Max), Confidence Interval (CI), Standard Error (Err Std). Created by the author.

## Discussion

The findings of this study represent a significant milestone in achieving consensus on therapeutic interventions for HF, with and without SCD, using the Delphi method with a panel of experts effectively reached consensus after three rounds of the process. These interventions encompassed cardiac resynchronization therapy with pacing (CRTp), cardiac resynchronization therapy with defibrillation (CRTd), implantable cardioverter-defibrillator (ICD), left ventricular assist device (LVAD), heart transplantation (CT), cardiac surgery, ablation, optimal medical treatment (OPT), non-pharmacological treatments (NPT), and other therapies for HF with and without SCD.

Comparing our findings with existing global literature, it is evident that the achieved consensus aligns with previous research in the field of HF management. Consensus on interventions such as CRTp, CRTd, and ICD is consistent with established guidelines and recommendations [19, 20]. Furthermore, the consensus in our study on LVAD as a therapeutic option is in line with studies highlighting its efficacy in advanced HF [21].

The composition of the diverse panel of experts in our study, including cardiologists and subspecialists with expertise in various aspects of HF management, reflects the recommendations of previous research. Prior studies have emphasized the importance of including participants with diverse perspectives and specialized knowledge to enhance the validity of consensus findings [22, 23].

It is crucial to emphasize that the primary goal of implementing clinical practice guidelines is to improve patient care, optimize HF management. The findings of our study, supported by the demonstrated clinical experience and knowledge of the panelists, contribute to the strength and relevance of the established consensus [24].

For future research, it is advisable to consider conducting larger studies involving the participation of multiple healthcare centers, both nationally and internationally. This approach could result in a higher response rate and may lead to substantial changes in the level of consensus achieved for the management of HF with and without SCD, in line with international variations and practices for this condition [25].

Additionally, as this study involved experts in cardiology with diverse perspectives on therapies for HF, the results may not be applicable to all care models for this condition. On occasion, respondents provided incomplete responses due to various reasons such as lack of financial incentives and workload. Achieving the optimal number of experts was a challenge, as there are no clear guidelines, although most studies suggest involving between 12 and 20 experts, we managed to involve 14 panelists.

## Conclusions

This study represents a significant advancement in developing clinical consensus on therapeutic interventions for HF in Colombia. Through the input and expertise of local healthcare professionals and experts in the field, consensus has been reached on 9 essential therapeutic interventions in the management of HF for the Colombian context, and other interventions have been identified, which experts recognize as part of the treatment, although they were not easy to identify in the guidelines and the review. These findings have the potential to improve the quality of care and clinical outcomes for patients with HF in the country. By establishing consensus among local experts, this study paves the way for a more standardized and evidence-based approach to HF treatment in Colombia, with the ultimate goal of improving the overall well-being of affected individuals.

It is essential to highlight that while this study provides valuable information on HF treatment in Colombia, its generalizability to other populations may be limited. The unique healthcare landscape, patient demographics, and healthcare infrastructure in Colombia can influence the relevance and applicability of these therapeutic interventions in other regions. Therefore, when considering the implementation of these findings in other contexts, it is crucial to consider the specific characteristics and needs of the local population. Future research and collaborations with experts from diverse backgrounds can help validate and adapt these consensus-based interventions for broader use.

Looking ahead, this study lays the groundwork for future research, policy development, and clinical practice improvement in the field of HF management and public health for individuals with HF. The identified therapeutic interventions provide a foundation upon which healthcare providers, policymakers, and researchers can build to enhance the standard of care for patients with HF not only in Colombia but in other Latin American countries facing similar challenges. As our understanding of HF and its management continues to evolve, insights gained from this study can contribute to more effective and patient-centered strategies to combat this prevalent and burdensome cardiovascular condition.

## Supporting information

S1 Appendix (DOCX)

S1 Table (XLSX)

S1 Data (XLSB)
