# Supplementary material for: Therapeutic interventions for heart failure in Colombia: result of a Delphi panel
Source: PLoS One. 2024 Sep 3;19(9):e0304124. doi: 10.1371/journal.pone.0304124 (PMC11371197; doi:10.1371/journal.pone.0304124)
Supplement: S1 Appendix — (DOCX) [file pone.0304124.s001.docx]

Appendix

Table 1. Definition of levels of consensus.

| **consensus level** | **General Practitioners (score > 8 out of 11 points on the Likert scale)** | **Cardiologist score < 8 out of 11 points on the Liker scale)** |
| --- | --- | --- |
| Perfect Consensus | 100% | 100% |
| Particularly good Consensus | 90% | 90% |
| \| Good Consensus \| \| --- \| | > 80% | > 70% |
| Some Consensus | > 60% | > 60% |
| No Consensus | All other cases | All other cases |

*Adapted from* (Verhestraeten et al., 2020) .
